# Differences and Similarities in Empathy Deficit and Its Neural Basis between Logopenic and Amnesic Alzheimer’s Disease

**DOI:** 10.3390/jpm13020208

**Published:** 2023-01-25

**Authors:** Giulia Giacomucci, Cristina Polito, Valentina Berti, Sonia Padiglioni, Giulia Galdo, Salvatore Mazzeo, Enrico Bergamin, Valentina Moschini, Carmen Morinelli, Claudia Nuti, Maria Teresa De Cristofaro, Assunta Ingannato, Silvia Bagnoli, Benedetta Nacmias, Sandro Sorbi, Valentina Bessi

**Affiliations:** 1 Department of Neuroscience, Psychology, Drug Research and Child Health, University of Florence, 50134 Florence, Italy; 2 IRCCS Fondazione Don Carlo Gnocchi, 50143 Florence, Italy; 3 Department of Biomedical, Experimental and Clinical Sciences “Mario Serio”, University of Florence, 50134 Florence, Italy; 4 Nuclear Medicine Unit, Azienda Ospedaliero-Universitaria Careggi, 50134 Florence, Italy; 5 Regional Referral Centre for Relational Criticalities—Tuscany Region, 50134 Florence, Italy; 6 Research and Innovation Centre for Dementia-CRIDEM, AOU Careggi, 50134 Florence, Italy; 7 University of Florence, 50134 Florence, Italy; 8 SOD Neurologia I, Dipartmento Neuromuscolo-Scheletrico e degli Organi di Senso, AOU Careggi, 50134 Florence, Italy

**Keywords:** Alzheimer’s disease, logopenic variant primary progressive aphasia, empathy

## Abstract

The aims of the study were to assess empathy deficit and neuronal correlates in logopenic primary progressive aphasia (lv-PPA) and compare these data with those deriving from amnesic Alzheimer’s disease (AD). Eighteen lv-PPA and thirty-eight amnesic AD patients were included. Empathy in both cognitive and affective domains was assessed by Informer-rated Interpersonal Reactivity Index (perspective taking, PT, and fantasy, FT, for cognitive empathy; empathic concern, EC, and personal distress, PD, for affective empathy) before (T0) and after (T1) cognitive symptoms’ onset. Emotion recognition was explored through the Ekman 60 Faces Test. Cerebral FDG-PET was used to explore neural correlates underlying empathy deficits. From T0 to T1, PT scores decreased, and PD scores increased in both lv-PPA (PT z = −3.43, *p* = 0.001; PD z = −3.62, *p* < 0.001) and in amnesic AD (PT z = −4.57, *p* < 0.001; PD z = −5.20, *p* < 0.001). Delta PT (T0–T1) negatively correlated with metabolic disfunction of the right superior temporal gyrus, fusiform gyrus, and middle frontal gyrus (MFG) in amnesic AD and of the left inferior parietal lobule (IPL), insula, MFG, and bilateral superior frontal gyrus (SFG) in lv-PPA (*p* < 0.005). Delta PD (T0-T1) positively correlated with metabolic disfunction of the right inferior frontal gyrus in amnesic AD (*p* < 0.001) and of the left IPL, insula, and bilateral SFG in lv-PPA (*p* < 0.005). Lv-PPA and amnesic AD share the same empathic changes, with a damage of cognitive empathy and a heightening of personal distress over time. The differences in metabolic disfunctions correlated with empathy deficits might be due to a different vulnerability of specific brain regions in the two AD clinical presentations.

## 1. Introduction

Empathy is widely recognized as the capacity to “put oneself in another’s shoes”, and it can be defined as the crucial ability to both feel and comprehend what others feel [1]. Empathy is conceptualized as a multidimensional construct regulated by unconscious affective and conscious cognitive processes [2]. Indeed, Decety and Jackson proposed the current model of empathy and suggested that empathy may be divided into two major components: affective empathy is the capacity to experience affective reactions to the observed experiences of others, while cognitive empathy is the capacity to recognize and understand another’s emotional state in order to enable the observer to adopt the other’s point of view [3]. 

Empathy seems to be impaired in several neurodegenerative diseases. Severe loss of empathy has been widely described as a diagnostic criterion of the behavioral variant of frontotemporal dementia (bv-FTD) [4,5,6]. On the other hand, despite studies that explored empathy deficit in Alzheimer’s disease (AD) not showing conclusive results [7], at the state of the art, researchers agree that empathy is impaired in AD, with a predominant loss of cognitive empathy, while the affective domain seems to be spared. Recently, it has been shown that there is a peculiar involvement of brain regions related to empathy in the AD continuum: the impairment of cognitive empathy seems to start at the mild cognitive impairment (MCI) stage and may be related to a progressive involvement starting from right middle frontal gyrus in the prodromal stage, extending to the insula and superior temporal gyrus in the dementia stage. On the other hand, an increase of emotional contagion (part of affective empathy) was already found in preclinical phases, and this alteration might be related to the derangement of mirror neurons systems in parietal regions in prodromal stages and to impairment of the temporal emotion inhibition system in advanced phases [8]. 

Despite the growing interest in empathy and social cognition in bv-FTD and A, little is known about the potential impairment of empathy in primary progressive aphasia (PPA). The term PPA defines a group of neurodegenerative syndromes characterized by progressive, selective decline in speech and language functions [9]. According to the current classification, three main clinical variants have been identified on the basis of specific linguistic features: non-fluent/agrammatic (nfv-PPA) and semantic (sv-PPA) variants, which are considered as part of the fronto-temporal lobar degeneration (FTLD) spectrum, and logopenic variant primary progressive aphasia (lv-PPA), which is considered as an atypical presentation of AD [9]. Interestingly, compromised brain regions across variants extend beyond classical language areas, and none of them is exclusively devoted to linguistic functions [10,11,12]. Deficits in both cognitive and affective empathy have been described in sv-PPA and may be independent from language dysfunction [13,14]. On the other hand, while affective empathy seems to be spared in nfv-PPA, findings about cognitive empathy are far from conclusive [15]. Finally, only few reports explore empathy and emotion recognition in lv-PPA, showing a decrease in cognitive empathy outcomes when compared to the pre-morbid results alongside a marginal reduction of affective empathy [15,16]. Evidence regarding empathy in lv-PPA is still sparse and inconclusive, and the neural correlates of empathy impairment have never been explored before. 

In this scenario, the aims of our study were: (1) to investigate possible empathy deficits in lv-PPA; (2) to explore the metabolic pattern associated with empathy deficit in lv-PPA by means of [18F]fluorodeoxyglucose positron emission tomography ([18F]FDG-PET); and (3) to compare empathy and its neural correlates in lv-PPA and in prototypical amnesic AD.

## 2. Materials and Methods

### 2.1. Participants

Eighteen patients with a clinical diagnosis of lv-PPA [9] and thirty-eight patients with a diagnosis of amnesic AD [17] were longitudinally included in this study. All participants underwent a comprehensive family and clinical history, general and neurological examination, extensive neuropsychological assessment, and evaluation of empathy through the Interpersonal Reactivity Index (IRI) [18,19]. Facial emotion recognition ability was assessed through the Ekman 60 Faces (EK-60 F) Test in 47 patients (34 AD and 13 lv-PPA) [20,21]. IRI and EK-60 F data of AD and lv-PPA patients were compared to those obtained in 31 subjects with subjective cognitive decline (SCD) [22]. Age at empathy assessment was defined as the age of the subject when the IRI and/or EK-60 F tests were administered. Age at onset was defined as age at the onset of complaints of cognitive symptoms. Seventy-one subjects underwent APOE genotyping. 

### 2.2. Neuropsychological Evaluation, Empathy, and Facial Emotion Recognition Assessment

All subjects were evaluated by means of an extensive neuropsychological battery standardized and described in further detail elsewhere [23]. Mini-Mental State Examination was used as global measurement. Short-term verbal and spatial memory were explored through Digit Span and Corsi Tapping Test and verbal long-term memory through Rey Auditory Verbal Learning Test, Immediate Recall RVLT-I and Delayed Recall RVLT-D, and Babcock Short Story Immediate and Delayed Recall. Token Test and Category Fluency Task were used to evaluate language [23,24,25]. Rey–Osterrieth Complex Figure copy and recall of Rey–Osterrieth Complex Figure test were used to explore visual-spatial abilities and visuo-spatial long-term memory, respectively [26]. Dual Task [27], Phonemic Fluency Test [25], Trail Making Test (TMT) [28], and Visual Search [29] were used to explore attention/executive function. Everyday memory was assessed by means of Rivermead Behavioral Memory Test (RBMT) [30]. All raw test scores were adjusted for age, education, and gender according to the correction factor reported in validation studies for the Italian population [23,24,26,27,28,29,30]. Language was further assessed by SAND (Screening for Aphasia in NeuroDegeneration) [31] or ENPA (Neuropsychological Evaluation for Aphasia) [32]. The presence and severity of depressive symptoms were evaluated by means of the 22-item Hamilton Depression Rating Scale (HRSD) [33].

Empathy was evaluated by Interpersonal Reactivity Index (IRI) [18,19], which consists of a 28-item questionnaire divided in four different 7-item subscales: perspective taking (PT), fantasy (FT), empathic concern (EC), and personal distress (PD). Perspective taking and fantasy subscales explore cognitive empathy, while empathic concern and personal distress subscales are used to assess affective empathy. In. more details, PD subscale is a measure of emotional contagion [34], which is a primitive structure of affective empathy and defined as the automatic, total identification with another’s behavior in order to encourage altruistic behavior [3]. Each item of IRI consists of an affirmation, and the individual has to express the degree of agreement on a 5-point Likert scale from 1 (does not describe me/the patient at all) to 5 (describes me/the patient very well). IRI was administered to the informants [35]. Caregivers had to rate patients’ empathy before (T0) and after (T1) cognitive symptoms’ onset. The differences from T0 to T1 of the scores of each scale were quantified as Delta (∆): ∆FT, ∆PT, ∆EC, and ∆PD. 

Facial emotion recognition was assessed by Ekman 60 Faces (EK-60 F) Test, which consists of 60 black and white pictures of the Ekman and Friesen series of Pictures of Facial Affect [20], representing the faces of ten actors (six women and four men), each of which shows one of six basic emotions (anger, sadness, happiness, fear, disgust, or surprise). A global score (EK-60 F global score) of 60 indicates the best possible performance. Each basic emotion has a sub-score of a maximum of 10 points. Images were shown each for 5 s according to the Ekman and Friesen procedure [20] via Power Point presentation on a computer. Patients were asked to indicate which of the basic emotions better represents the facial emotion shown on the display [21]. 

### 2.3. Apolipoprotein E (APOE) Genotyping

Patients’ DNA was extracted from peripheral blood samples by use of a standard automated method (QIAcube, QIAGEN Hilden, Germany). APOE genotypes were investigated by HRMA [36]. Two sets of PCR primers were designed to amplify the regions encompassing rs7412 (NC_000019.9:g.45412079C > T) and rs429358 (NC_000019.9:g.45411941T > C). The APOE genotype was coded as APOE ε4− (no APOE ε4 alleles) and APOE ε4+ (presence of one or two APOE ε4 alleles).

### 2.4. Amyloidosis and Neurodegeneration Biomarkers Analysis

Amyloidosis biomarkers analysis was performed in 69 patients. Sixty-four patients (27 amnesic AD, 17 lv-PPA, 20 SCD) underwent cerebrospinal fluid (CSF) biomarkers analysis.

The CSF samples were collected at 8 a.m. by lumbar puncture, immediately centrifuged, and stored at −80 °C until performing the analysis. Aβ1-42, Aβ1-42/1-40 ratio, t-tau, and p-tau were measured using a chemiluminescent enzyme immunoassay (CLEIA) analyzer LUMIPULSE G600 (Fujirebio). Fujirebio guidelines determined cut-off values (Diagnostic sensitivity and specificity using clinical diagnosis and follow-up golden standard. 19 November 2018), which were Aβ1–42 > 670 pg/mL, Aβ42/40 ratio > 0.062, t-tau < 400 pg/mL, and p-tau < 60 pg/mL [37]. 

Twenty-three patients (7 amnesic AD, 6 lv-PPA, 10 SCD) underwent cerebral amyloid PET. Amyloid PET imaging was performed according to national and international guidelines [38], with any of the available fluorine18-labeled tracers (18Florbetaben (FBB)—Bayer-Pyramal, 18Flutemetamol (FMM)—General Electric). Images were rated as either positive or negative according to criteria defined by the manufacturers. 

According to ATN classification [39], patients were classified as A+ if at least one of the amyloid biomarkers (CSF or amyloid PET) revealed the presence of Aβ pathology and as A- if none of the biomarkers revealed the presence of Aβ pathology. All amnesic AD and lv-PPA patients were A+.

### 2.5. FDG-PET Brain Imaging

All amnesic AD and lv-PPA patients underwent brain [18F]FDG-PET. Scans were performed using advanced hybrid PET-CT scanner in 3D list mode. Patients were instructed to fast for 6 h before the study, and blood sugar level was tested to be lower than 120 mg/dL. Patients were injected with 185 MBq of [18F]-FDG via a venous cannula. After the injection, patients were left in a dimly lit, quiet room and told to keep their eyes closed. All [18F]FDG-PET scans were acquired following the EANM guidelines [40]. All PET images were corrected for photon attenuation, scatter, and radioactive decay and reconstructed using 3D iterative algorithm. [18F]FDG-PET scans pre-processing and statistical analysis are described in Section 2.6.

### 2.6. Statistical Analysis

IBM SPSS Statistics Software Version 25 (SPSS Inc., Chicago, IL, USA) and computing environment R 4.0.3 (R Foundation for Statistical Computing, Vienna, 2013) were used to perform all statistical analysis. All p-values were two-tailed, and the significance level for all analyses was set at α = 5%, corresponding to a threshold *p* of 0.05. All variables are described as mean and standard deviation. Shapiro–Wilk test was used to assess the distribution of all variables. Chi-square test was used to compare categorical data. One-way ANOVA followed by Bonferroni post hoc test was used to evaluate differences among groups in continuous variables. Variation of IRI scores over time, from before to after the onset of cognitive symptoms (T0-T1), was explored through Wilcoxon signed-rank test. The influence of demographic variables and neuropsychological scores on current empathy and on facial emotion recognition ability was investigated through Spearman’s correlation. Bonferroni correction for multiple comparisons was applied for correlations between each IRI subscales and demographic features (*p* = 0.0019), neuropsychological measures (*p* = 0.0019), and SAND scores (*p* = 0.0016); similarly, it was applied for correlations between facial emotion recognition ability and demographic features (*p* = 0.002), neuropsychological measures (*p* = 0.002), and SAND scores (*p* = 0.0017). 

### 2.7. SPM Analysis

In order to assess the metabolic pattern related to empathy changes in lv-PPA and how it differs between prototypical amnesic AD and lv-PPA, a total of 42 patients were considered (26 amnesic AD and 16 lv-PPA patients). Each patient had positive amyloid biomarkers. [18F]FDG-PET images were normalized to the MNI space using a validated procedure. Images were smoothed with an isotropic 3D Gaussian kernel with a FWHM of 8 mm in each direction and then were used for a single-subject SPM-based routine for diagnostic purposes [41]. Age was included as a covariate in the two-sample *t*-test analysis. The correlation between IRI subscales, resulting from behavioral data analysis, and brain hypometabolism in the amnesic AD and lv-PPA groups was explored using the SPM multiple regression design, including age and MMSE as nuisance variables in the linear model. The threshold was set at *p*-value < 0.001, uncorrected, to test for correlations also in the small subsamples of amnesic AD and lv-PPA. Only clusters containing more than 50 voxels were considered significant. 

### 2.8. Data Availability

The data that support the findings of this study are available from the corresponding author upon reasonable request.

## 3. Results

### 3.1. Demographic Features and Biomarkers Analysis

Table 1 shows the demographic variables of the cohort. Considering the whole sample, 57 patients were females and 30 males. Age at onset was significantly different among the three groups (F [2, 82] = 17.527, *p* < 0.001): indeed, SCD (54.40 ± 10.08, *p* < 0.001) were younger than amnesic AD (66.53 ± 6.708, *p* < 0.001) and lv-PPA (64.50 ± 7.60, *p* < 0.001) patients. Age at empathy assessment was significantly different among groups (F [2, 83] = 3.739, *p* = 0.028): in detail, at empathy evaluation, SCD were younger (65.61 ± 9.48) than amnesic AD (71.04 ± 7.33, *p* < 0.024) but not younger than lv-PPA (68.65 ± 7.02, *p* = 1.000) patients. Mini Mental State Examination (MMSE) was different among the groups (F [2, 81] = 48.897 *p* < 0.001), with lower scores in amnesic AD (17.03 ± 5.28) as compared to SCD (27.73 ± 2.04, *p* < 0.001) but not to lv-PPA (16.69 ± 6.37, *p* = 1.000) patients. Considering the subsample who underwent APOE genotype analysis, 36.62% resulted to be APOE ε4 carriers.

Sixty-four patients (27 amnesic AD, 17 lv-PPA, 20 SCD) underwent CSF biomarkers analysis. Twenty-three patients (7 amnesic AD, 6 lv-PPA, 10 SCD) were subjected to cerebral amyloid PET, which was positive in 17 patients (6 amnesic AD, 6 lv-PPA, 5 SCD). Based on the positivity for at least one cerebral amyloidosis biomarker, 30 amnesic AD and 18 lv-PPA patients and 8 subjects with SCD were classified as A+ (56 out of 69, 82.35%). 

### 3.2. IRI Empathy Results

Significant differences were detected neither in premorbid empathy, in IRI total score T0 (F [2, 79] = 0.397, *p* = 0.674), nor in any subscale FT-T0 (F [2, 79] = 1.599, *p* = 0.209), PT-T0 (F [2, 79] = 1.945, *p* = 0.150), EC-T0 (F [2, 79] = 0.530, *p* = 0.590), and PD-T0 (F [2, 79] = 1.014, *p* = 0.368) among the three groups. 

**Figure 1:**
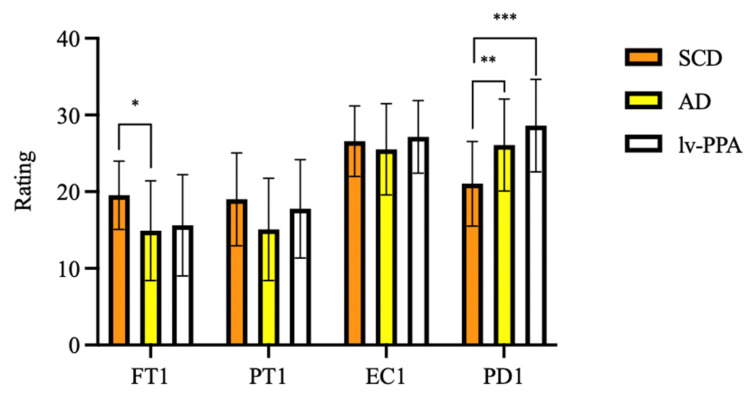
Empathy assessed by Interpersonal Reactivity Index (IRI) in subjective cognitive decline (SCD), Alzheimer’s disease (AD) and logopenic variant primary progressive aphasia (lv-PPA). * *p* < 0.05, ** *p* < 0.005, *** *p* < 0.001.

As regards current empathy, one-way ANOVA showed significant differences in FT-T1 (F [2, 79] = 5.046, *p* = 0.008) and PD-T1 (F [2, 79] = 10.004, *p* < 0.001) among groups. At Bonferroni post hoc test, amnesic AD patients’ FT-T1 scores were significantly lower than those of SCD (14.91 ± 6.50 vs. 19.54 ± 4.45, *p* = 0.009). Both lv-PPA (28.62 ± 6.03, *p* < 0.001) and amnesic AD (26.09 ± 6.00, *p* = 0.003) presented higher scores than SCD (21.04 ± 5.51) on the PD-T1 subscale, while no differences were found between lv-PPA and amnesic AD patients (*p* = 0.481). No significant differences were found in PT-T1 and EC-T1 scores among groups (Figure 1). 

To estimate changes of empathy from before to after the onset of cognitive symptoms in amnesic AD and lv-PPA, Wilcoxon signed-rank test was used (Table 2). A significant decrease of FT (17.11 ± 5.09 vs. 15.05 ± 6.21, z = −2.464, *p* = 0.014) was detected in amnesic AD subgroup. A decrease in PT scores was found both in amnesic AD (21.08 ± 6.15 vs. 15.42 ± 6.66, z = −4.753, *p* < 0.001) and in lv-PPA patients (23.94 ± 6.40 vs. 15.82 ± 6.61, z = −3.435, *p* < 0.001). Finally, a significant increase of PD was found both in amnesic AD (17.08 ± 4.99 vs. 26.03 ± 5.72, z = −5.204, *p* < 0.001) and in lv-PPA patients (15.76 ± 5.47 vs. 28.35 ± 6.07, z = −3.623, *p* < 0.001). 

### 3.3. EK-60 F Emotion Recognition Results

Considering emotion recognition ability assessed by EK-60F test, all the variables were significantly different among the three groups (Table 3). At EK-60F global score, lv-PPA and amnesic AD patients performed significantly poorer than SCD (32.25 ± 8.09 vs. 33.29 ± 7.56 vs. 47.58 ± 5.14, respectively, *p* < 0.001). Concerning the single emotions’ recognition, lv-PPA and amnesic AD had worse performances in the recognition of anger, disgust, sadness, surprise, and happiness detection as compared to SCD (Table 3). 

**Figure 2:**
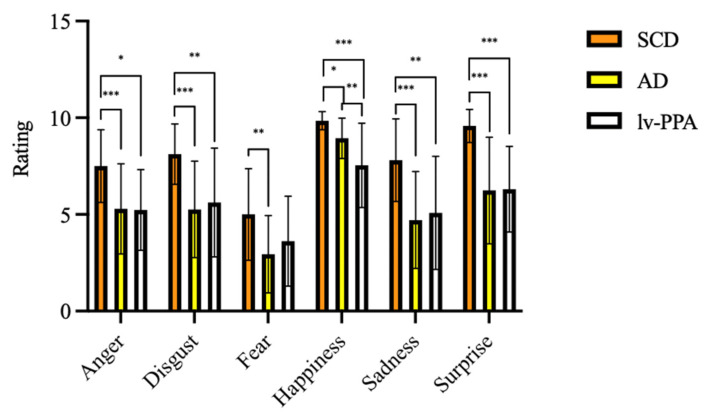
Emotional recognition ability as assessed by Ekman 60 Faces Test in subjective cognitive decline (SCD), Alzheimer’s disease (AD), and logopenic variant primary progressive aphasia (lv-PPA). * *p* < 0.05, ** *p* < 0.005, *** *p* < 0.001.

SCD scores for fear recognition were higher than those obtained by amnesic AD patients (5.10 ± 2.68 vs. 2.94 ± 2.00, *p* = 0.001) but not higher than those obtained by lv-PPA (3.67 ± 2.43, *p* = 0.235). On the other hand, no differences were detected between lv-PPA and amnesic AD patients except for happiness, with lower scores found in the lv-PPA subgroup as compared to the amnesic AD one (7.33 ± 2.15 vs. 8.94 ± 1.04 vs., *p* = 0.01) (Figure 2).

### 3.4. Correlations between Demographic Data, Neuropsychological Variables, Empathy, and Emotion Recognition

Correlations were found neither between each IRI subscales, age at empathy evaluation, years of education, and HDRS scores; nor in the whole cohort; nor in lv-PPA; nor in amnesic AD subgroups. On the other hand, significant differences between women and men in the whole group were found: women had higher scores than men in IRI-T0 (84.02 ± 11.69 vs. 78.46 ± 10.71, *p* = 0.034), IRI-T1 (86.67 ± 11.88 vs. 79.79 ± 13.94, *p* = 0.044), FT-T0 (18.45 ± 4.74 vs. 16.54 ± 4.85, *p* = 0.031), FT-T1 (17.86 ± 5.56 vs. 14.29 ± 6.79, *p* = 0.004), and PD-T0 (21.16 ± 6.29 vs. 20.92 ± 5.36, *p* = 0.024). These gender differences were not found when we analyzed lv-PPA and amnesic AD except for IRI-T1 scores, which were higher in amnesic AD women as compared to men (84.29 ± 14.04 vs. 77.53 ± 11.69, *p* = 0.021). We also analyzed correlations between neuropsychological tests, SAND evaluation, and each IRI subscale: correlations were found neither in the whole cohort, nor in lv-PPA, nor in amnesic AD subgroups.

Correlations were found between each EK-60 F scores, age at empathy evaluation, and years of education neither in the whole cohort, nor in lv-PPA, nor in amnesic AD subgroups. Similar to IRI subscales, we found significant difference between women and men in the whole group: in more detail, women obtained higher scores as compared to men in EK-60 F total (40.16 ± 9.92 vs. 34.75 ± 8.40, *p* = 0.012), disgust (6.20 ± 2.50 vs. 5.17 ± 2.46, *p* = 0.004), sadness (6.45 ± 2.65 vs. 4.71 ± 2.88, *p* = 0.011), and surprise (7.86 ± 2.53 vs. 6.50 ± 2.80, *p* = 0.017). Moreover, EK-60 F execution time was lower in women as compared to men (361.69 ± 121.13 vs. 438.13 ± 110.53, *p* = 0.001). These gender differences were not found when we analyzed lv-PPA and amnesic AD subgroups. Moreover, no correlations were found between neuropsychological tests, SAND evaluation, and emotion recognition ability (both EK-60 F total score and single emotion recognition scores) in the whole cohort, in logopenic, or in amnesic Alzheimer’s disease subgroups.

### 3.5. SPM Results

Significant correlations between IRI subscales and brain metabolism in amnesic AD and lv-PPA groups were found through the SPM multiple regression analysis. 

**Figure 3:**
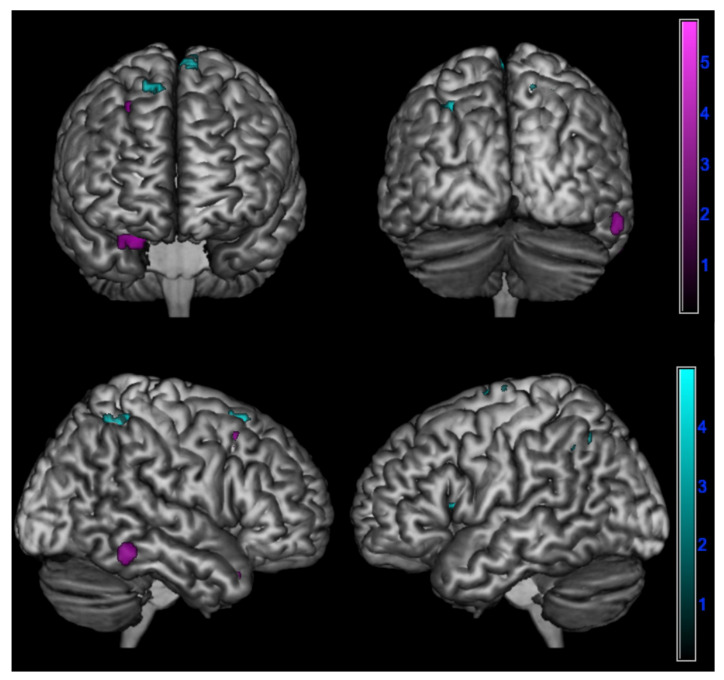
Negative correlation between changes in perspective taking ability over time assessed by ∆PT (PT-T0 - PT-T1) and brain metabolism in logopenic primary progressive aphasia and amnesic Alzheimer’s disease patients at 18F-FDG-PET SPM analysis. Significant clusters projected on the standardized Montreal Neurological Institute (MNI) magnetic resonance imaging (MRI) render surface. Color grading: cyan, lv-PPA; violet, amnesic AD.

In detail, a negative correlation between ∆PT and brain metabolism was found:

- In amnesic AD in the right superior temporal gyrus, fusiform gyrus, and middle frontal gyrus (*p* < 0.005);
- In lv-PPA in the left inferior parietal lobule, left insula, left middle frontal gyrus, right posterior paracentral lobule, and bilateral superior frontal gyrus (*p* < 0.005) (Table 4 and Figure 3).

**Figure 4:**
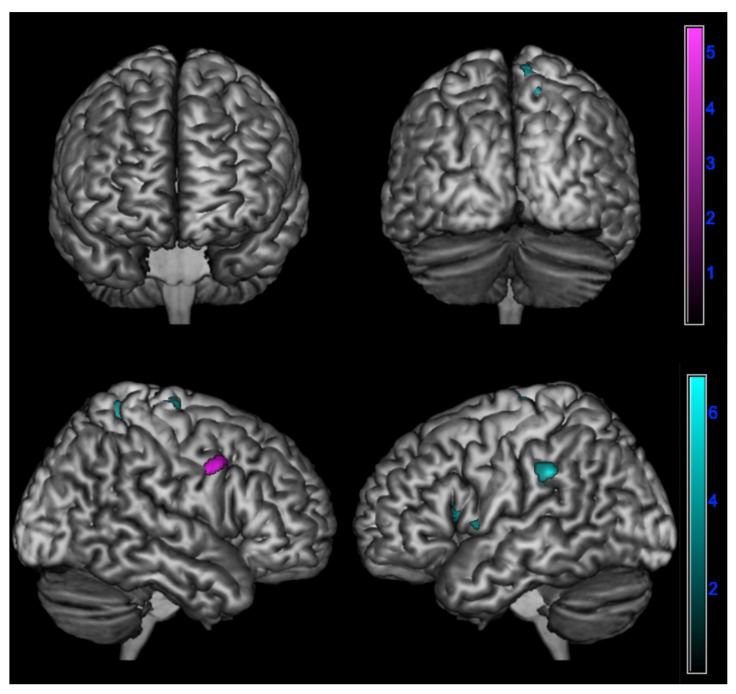
Positive correlation between changes in emotional contagion over time assessed by ∆PD (PD-T0 - PD-T1) and brain metabolism in logopenic primary progressive aphasia and amnesic Alzheimer’s disease patients at 18F-FDG-PET SPM analysis. Significant clusters projected on the standardized Montreal Neurological Institute (MNI) magnetic resonance imaging (MRI) render surface. Color grading: cyan, lv-PPA; violet, amnesic AD.

A positive correlation between ∆PD and brain metabolism was found:

- In amnesic AD in the right inferior frontal gyrus (*p* < 0.001);
- In lv-PPA in the left inferior parietal lobule, insula, and superior frontal gyrus and in the right precuneus and superior frontal gyrus (*p* < 0.005) (Table 4 and Figure 4).

## 4. Discussion

Our study is the first that deeply explored empathy in lv-PPA while also trying to define the neural correlates of empathy impairment in this variant of PPA, which is considered as an atypical presentation of Alzheimer’s disease. Moreover, for the first time, we compared the behavioral and metabolic data of empathy between lv-PPA and prototypical amnesic AD in order to define similarities and differences between these two presentations of Alzheimer’s disease.

Our study showed several similarities in empathic impairment between lv-PPA and amnesic AD. In more detail, both lv-PPA and amnesic AD patients presented a decrease in PT over time, suggesting a significant damage to cognitive empathy. On the other hand, a relative sparing of EC (part of affective empathy) was found in both subgroups. 

In particular, a significant decline in PT was found in both logopenic and in amnesic AD, suggesting that both clinical phenotypes of AD present a significant change of cognitive empathy from before to after the onset of cognitive symptoms. Although results about empathy deficits in AD are far from conclusive, recent studies suggested a selective impairment of cognitive empathy [35,42,43,44]. Moreover, a decrease of perspective taking ability from before to after the onset of cognitive disturbance has been recently described in AD, and this change seems to be present also in the prodromal stage of the disease [8]. Considering lv-PPA, Hazelton et al. already reported a disruption of cognitive empathy, showing a decrease of perspective taking ability by comparing premorbid and present scores of the PT subscale [15]. 

Affective empathy seems to be spared both in lv-PPA and in amnesic AD patients since no significant changes were found in EC scores from before to after the onset of cognitive symptoms. Moreover, no differences were found in EC-T1 among lv-PPA, amnesic AD, and also SCD patients, which may be considered as the preclinical phase of cognitive decline [45]. However, we found changes in PD in both lv-PPA and in amnesic AD. Personal distress has been widely defined as a measure of emotional contagion, which could be considered as a primitive structure of emotional empathy and indicates the tendency to automatically adopt the behavior of another person [2]. Both lv-PPA and amnesic AD patients presented higher PD scores as compared to SCD. When we analyzed the trend of each component of empathy over time, we found a significant increase in PD from before to after the onset of cognitive disturbs both in amnesic AD and in lv-PPA patients, leading to the further hypothesis that emotional contagion heightens with the development of cognitive decline. 

Concerning amnesic AD, our results are in line with some studies that previously described a higher emotional contagion as compared to healthy controls [7,34]: Sturm et al. hypothesized that emotional contagion might increase linearly from healthy controls to MCI and AD patients, who presented the highest degree of emotional contagion [34]. Moreover, a previous work of our group has already shown a significant increase of PD scores from before to after the onset of cognitive disturbance [8], suggesting that this change in emotional contagion might be a peculiar feature of AD patients. 

Changes in PD scores in PPA have also been previously described by Hazelton et al., who explored empathy deficit in lv-PPA and nfv-PPA, showing an increase in personal distress in both variants following the disease onset [15]. 

We also explored emotion recognition ability through the EK-60 F test. According to our results, both lv-PPA and amnesic AD patients presented difficulties in facial emotion recognition, showing lower scores in recognition of almost all six basic emotions as compared to SCD subjects. The only exception was the recognition of fear since no differences were detected between lv-PPA and SCD subgroups, while amnesic AD patients performed more poorly than SCD. Our results are in line with the current literature reporting severe difficulties in identification of facial emotions in AD patients [46,47,48,49]. On the other hand, emotion recognition has been only recently studied in lv-PPA, and results are far too few to be clearly conclusive. A previous study reported spared performance in this variant through a task requiring identification of emotional expressions in static faces [16]. On the other hand, another work reported better performance in an emotion recognition task involving naturalistic scenes (i.e., TASIT) in lv-PPA than nfv-PPA, in which emotion recognition seems to be impaired [15,16,50]. Nevertheless, this difference was not statistically significant. Moreover, it has been shown that lv-PPA significantly outperforms those with semantic variant [51]. Thus, considering these findings, it has been hypothesized that emotion recognition deficits in lv-PPA (if present) may be milder than in other variants [10]. However, considering our results, we might suggest that emotion recognition ability may be impaired in lv-PPA. However, these data need to be confirmed.

Globally, no clear differences were detected in emotion recognition ability between lv-PPA and amnesic AD. Although several studies reported incongruent results, emotion recognition ability seems to be impaired in AD [8,47,48]. Indeed, it has been suggested that the recognition of specific emotions (i.e., disgust) may be spared in AD probably due to the relative sparing of basal ganglia [52]. However, these findings have not been confirmed in other works [8]. To the best of our knowledge, no previous studies have analyzed this aspect by comparing these two presentations of Alzheimer’s disease. Our results lead to the hypothesis that this emotion processing function is similarly impaired in the two presentation of the disease. Notably, the only difference was found in the recognition of happiness, with more difficulties found in lv-PPA as compared to amnesic AD patients. This result is challenging to discuss. In fact, this finding has never been previously described. Piguet et al. analyzed emotion recognition ability through the EK 60-F test by comparing healthy controls, nfv-PPA, and lv-PPA patients, showing significant group differences for the negative (anger, disgust, fear, and sadness) but not the positive (surprise and happiness) emotions due to the low performance of the non-fluent (but not logopenic) group compared to healthy controls. In addition, non-fluent patients also performed worse than logopenic patients for the emotion fear, which is considered as a negative emotion [16]. Such difficulties in happiness recognition in our lv-PPA patients have never been described so far: this subtle difference may be highlighted in our work through the comparison between the two presentation of AD. However, this result needs to be confirmed in further studies with larger cohorts.

Interestingly, we did not find any correlation between demographic data, neuropsychological tests, language evaluation, IRI subscale, and emotion recognition in either lv-PPA or in amnesic AD patients. This is partially in contrast with a previous report according to which the reduction of PT scores in lv-PPA was correlated with visuospatial abilities [15]. However, no further studies have explored this topic yet as well as correlations between emotion recognition ability and neuropsychological evaluation. Moreover, it has been recently highlighted that PD positively correlates with depression in healthy subjects [53]: indeed, no correlations were found between HDRS and PD in our cohort either in lv-PPA or in amnesic AD patients. Although our work is a preliminary study, our results might suggest that the empathic impairment and difficulties in emotion recognition might not be correlated with cognitive decline, with depressive symptoms, and in particular with language impairment in lv-PPA patients. Nevertheless, further studies are needed to confirm this hypothesis. 

The further aims of our study were to explore the metabolic pattern associated with empathy changes in lv-PPA and to compare empathy and its neural correlates in logopenic and in prototypical amnesic AD. First of all, to the best of our knowledge, this is the first study that explored the neural bases of empathy deficits in lv-PPA. Surprisingly, despite the similar empathy impairment, the metabolic correlates of empathy deficits were different between lv-PPA and amnesic AD patients.

Indeed, while in amnesic AD patients, the empathic dysfunction was related to hypometabolism of specific brain regions mainly located on the right hemisphere, in lv-PPA, it was correlated with hypometabolism of both hemispheres. This involvement of both hemispheres in lv-PPA might be explained by the fact of the presence of a specific and peculiar left lateralization of neurodegeneration in this disease [9].

Cognitive empathy changes over time were correlated with the involvement of the right superior temporal gyrus, middle frontal gyrus, and fusiform gyrus in amnesic AD and with involvement of the left inferior parietal lobule, insula, middle frontal gyrus, and bilateral superior frontal gyrus in lv-PPA. 

Previous works have already described the correlation between cognitive empathy deficits and the involvement of superior temporal and middle frontal gyri in AD patients [8]. This correlation might be explained by the fact that superior temporal gyrus plays a role in mentalizing activity and perspective-taking tasks [54,55] through its connections with the temporal poles and medial prefrontal cortex [56]. Similarly, the middle frontal gyrus, as part of the dorsolateral prefrontal cortex (DLPFC), is involved perspective-taking tasks [55,57]. Moreover, it seems to intentionally inhibit self-perspective in order to consider the other’s point of view [35] and to be involved in emotion evaluation [58]. Interestingly, we also found an involvement of the fusiform gyrus: Rankin et al. already described the role of the fusiform gyrus in cognitive empathy, in particular in perspective-taking ability, probably related to its direct involvement in facial perception and recognition [35]. 

On the other hand, loss of perspective taking over time in lv-PPA has been related to the impairment of the inferior parietal lobule, insula, middle frontal gyrus, and superior frontal gyrus. As regards the inferior parietal lobule, although it has been described as directly involved in the network of emotional contagion, it has been clearly stated that perspective-taking tasks engage several brain regions that also include the inferior parietal lobule [55]. Similarly, the superior frontal gyrus, in particular the supplementary motor area, seems to be involved in cognitive empathy tasks [55]. Concerning the insula, it is well-known that this brain region plays a major role in generating forward models of feeling states for others in order to predict and understand the social and affective behavior of other people [2,55,59]. 

Such neurophysiological bases might explain the association of the impairment of these brain regions and the perspective-taking deficits over time. Nevertheless, our findings might suggest that loss of perspective-taking ability over time might be related to the selective impairment of different empathy-related brain regions in the two clinical presentation of Alzheimer’s disease.

We also found that the amplification of emotional contagion over time was correlated with the involvement of the inferior parietal lobule, insula, superior frontal gyrus, and precuneus in lv-PPA and with the involvement of the right inferior frontal gyrus in prototypical amnesic AD. The involvement of frontal and parietal regions (in particular of the inferior frontal gyrus and inferior parietal lobule) may be explained by the fact that these areas are part of the mirror neurons system (MNS) network [2]. Emotional contagion is rooted in the MNS, which transforms sensory representations of others’ behavior into one’s own visceromotor representations and allows understanding others’ actions according to the perception–action model [59]. In more detail, it has been suggested that the inferior frontal gyrus identifies the goals or intentions of actions by their resemblance to stored representations for these actions [60]. Despite the unquestioned involvement in cognitive empathy, several studies have also described a putative role of the insula in the mirror mechanism of emotions: in fact, it has been demonstrated that there is a clear overlap between insular activation elicited by one’s own and others’ emotions, such as disgust [59,61,62]. Similarly, the insula is also involved in the empathic process of “shared pain”: in fact, empathizing with people in pain is associated with hemodynamic activity in the brain that is similar to the activity that occurs when people feel pain themselves, leading to the activation of the “empathy to pain network”, which involves the anterior cingulate cortex and insula [63]. It has been suggested that empathy for pain of others may be considered, at least in part, as an automatic, primitive, bottom-up process of affective empathy since it may aid in the immediate perception and avoidance of a threat to oneself [64]. The superior frontal gyrus and supplementary motor area also seem to be involved not only in cognitive empathy tasks but also in affective empathy [55].

The most interesting data show that similar changes in empathy correspond to the involvement of different brain regions in the two phenotypes of Alzheimer’s disease. This may be explained by the fact that empathy is a complex construct: a cognitive function that implies several interconnected brain regions [3]. On the other hand, it is well-known that neurodegeneration in lv-PPA and in prototypical amnesic AD involve different brain regions due to the selectivity of vulnerability of these two presentations [65,66]. Considering these premises, we might hypothesize that the selective neurodegeneration of the two clinical subtypes of Alzheimer’s disease might damage different brain regions and networks related to empathy, leading to the same impairment. 

Our study has some remarkable strengths. First of all, this is one of the first studies deeply analyzing empathy changes in lv-PPA and comparing empathy impairment with that found in amnesic AD patients in order to detect differences and similarities between these two clinical presentations of the disease. Another strength is the research of the neural correlates of empathy deficits in lv-PPA, which have never been explored before, by means of FDG-PET.

The main limitation of our study is the relatively small sample size and thus the lack of corrections for multiples comparisons in the correlation analysis between empathy deficits and hypometabolism in FDG PET analysis. Indeed, we chose to use a more exploratory threshold in order to explore the metabolic correlates of empathy also in the small subsamples of the lv-PPA and amnesic AD patients individually considered. Another limitation of the study is the use of a caregiver-report questionnaire even though the IRI is the most used, validated instrument for the evaluation of empathy. In fact, even if observer-based measures are more ecologically valid and have yielded valuable data previously [67], they are nevertheless limited by their dependence on informants’ varying reliability [68]. On the other hand, the scores attributed by the informants present the advantage of capturing real-life empathic behavior independently from the patients’ anosognosia [10]. The last limitation is the absence of healthy controls and the need to use SCD to compare the behavioral data although despite this category of subjects has been already used in previous work exploring empathy in AD [69].

## 5. Conclusions

In conclusion, to the best of our knowledge, our study analyzed for the first time differences and similarities between lv-PPA and amnesic AD, which are two clinical presentations of this neurodegenerative disease. We described a peculiar involvement of specific brain areas in empathy changes in lv-PPA, comparing them with that found in amnesic AD.

Interestingly, lv-PPA and amnesic AD presented the same changes in empathy, showing a damage of perspective-taking ability (part of cognitive domain) together with a heightening of emotional contagion (the most primitive structure of affective empathy) over time. Nevertheless, these similar changes in empathy correspond to the involvement of different brain regions in the two phenotypes of Alzheimer’s disease. This could be explained by the fact that neurodegeneration might damage different brain regions and networks related to empathy in these two presentations of Alzheimer’s disease due to the selectivity of vulnerability of the brain areas of these variants.

Moreover, our study described empathy changes and difficulties in emotion recognition in lv-PPA. In fact, while clinical diagnosis of PPA is basically anchored in linguistic disturbances, a proportion of cases cannot be easily classified based solely on language tests. In this scenario, emerging experimental works are revealing impairments in several socio-cognitive processes in these patients in association with brain measures. While some studies have already described deficits in emotion recognition, the theory of mind, and empathy in nfv-PPA and sv-PPA, evidence about lv-PPA are sparse and far from conclusive. Our work showed for the first time that lv-PPA patients presented deficits in empathy and in emotion recognition, too. Further studies are needed to confirm our results and better explore these socio-cognitive aspects of PPA, which might be very helpful in improving early diagnostic accuracy.

## Figures and Tables

**Table 1:** Demographic features in Alzheimer’s disease (AD) and logopenic variant primary progressive aphasia (lv-PPA) groups.

	SCD *n* = 31	AD *n* = 38	lv-PPA *n* = 18
**Gender (M/F)**	4/27 * ^α^	17/21 *	9/9 ^α^
**Age at onset (years)**	54.94 ± 10.08 ^βγ^	66.53 ± 6.71 ^β^	64.50 ± 7.60 ^γ^
**Age at empathy (years)**	65.61 ± 9.48 ^δ^	71.04 ± 7.33 ^δ^	68.65 ± 7.02
**Disease duration (years)**	9.62 ± 7.51 ^εη^	4.44 ± 3.59 ^ε^	2.87 ± 1.54 ^η^
**Family history of dementia**	22/6 ^θ^	16/17 ^θ^	6/11
**Years of education**	12.58 ± 3.40 ^ι^	9.80 ± 4.65 ^ικ^	13.22 ± 4.45 ^κ^
**MMSE**	27.43 ± 2.04 ^λ^ * ^μ^ *	17.03 ± 5.28 ^λ^	16.69 ± 6.37 * ^μ^ *
***APOE* ε4+**	30.77%	50.00%	50.00%
**A+/A−**	8/12 (40%) ^ν°^	30/0 (100%) ^ν^	18/0 (100%) ^°^

Values are reported as mean and standard deviation for continuous variables and as frequencies or percentages for categorical variables. Statistical differences among groups are underlined. M, males; F, females; MMSE, Mini Mental State Examination. * χ^2^ = 8.172, *p* = 0.008; α χ^2^ = 6.126, *p* = 0.019; β *p* < 0.001; γ *p* = 0.001; δ *p* = 0.024; ε *p* < 0.001; η *p* < 0.001; θ χ^2^ = 5.838, *p* = 0.019; ι *p* = 0.025; κ *p* = 0.018; λ *p* < 0.001; μ *p* < 0.001; ν χ^2^ = 24.92, *p* < 0.001; ° χ^2^ = 16.71, *p* < 0.001.

**Table 2:** Change of empathy capacity from before to after the onset of cognitive symptoms in Alzheimer’s disease (AD) and logopenic variant primary progressive aphasia (lv-PPA) groups.

	AD			lv-PPA		
	Mean ± SD	*z*	*p*	Mean ± SD	*z*	*p*
IRI 0 IRI 1	81.26 ± 13.31 81.95 ± 13.35	−0.828	0.408	83.41 ± 11.12 83.76 ± 14.90	−0.166	0.868
FT 0 FT 1	17.11 ± 5.09 15.05 ± 6.21	−2.464	**0.014**	17.18 ± 5.89 15.24 ± 6.21	−1.574	0.115
PT 0 PT 1	21.08 ± 6.15 15.42 ± 6.66	−4.752	**<0.001**	23.94 ± 6.40 15.82 ± 6.61	−3.435	**0.001**
EC 0 EC 1	26.39 ± 4.45 25.76 ± 5.70	−1.185	0.236	25.94 ± 5.12 24.35 ± 6.47	−1.250	0.211
PD 0 PD 1	17.08 ± 4.99 26.03 ± 5.72	−5.204	**<0.001**	15.76 ± 5.47 28.35 ± 6.07	−3.623	**<0.001**

Values are reported as mean and standard deviation. Statistical differences between groups are in **bold**.

**Table 3:** Facial emotion recognition ability in subjective cognitive decline (SCD), Alzheimer’s disease (AD), and logopenic variant primary progressive aphasia (lv-PPA) as assessed by the Ekman 60 Faces Test (EK-60 F).

	SCD	AD	lv-PPA	F	*p*	*p* between SCD and AD	*p* between SCD and lv-PPA	*p* between AD and lv-PPA
**EK-60 F total score**	47.58 ± 5.14	33.29 ± 7.56	32.25 ± 8.09	42.863	***p* < 0.001**	***p* < 0.001**	***p* < 0.001**	*p* = 1.000
**Execution time (seconds)**	290 ± 56.19	445.74 ± 124.03	425.08 ± 103.60	21.427	***p* < 0.001**	***p* < 0.001**	***p* < 0.001**	*p* = 1.000
**Anger**	7.35 ± 1.80	5.29 ± 2.33	5.08 ± 2.11	9.480	***p* < 0.001**	***p* = 0.001**	***p* = 0.006**	*p* = 1.000
**Disgust**	8.10 ± 1.45	5.26 ± 2.49	5.25 ± 2.60	16.134	***p* < 0.001**	***p* < 0.001**	***p* = 0.001**	*p* = 1.000
**Fear**	5.10 ± 2.68	2.94 ± 2.00	3.67 ± 2.43	6.862	***p* = 0.002**	***p* = 0.001**	*p* = 0.235	*p* = 1.000
**Happiness**	9.84 ± 0.45	8.94 ± 1.043	7.33 ± 2.15	21.962	***p* < 0.001**	***p* = 0.006**	***p* < 0.001**	***p* < 0.001**
**Sadness**	7.97 ± 2.01	4.71 ± 2.51	4.75 ± 2.80	17.476	***p* < 0.001**	***p* < 0.001**	***p* < 0.001**	*p* = 1.000
**Surprise**	9.48 ± 1.03	6.24 ± 2.75	6.17 ± 2.25	21.666	***p* < 0.001**	***p* < 0.001**	***p* < 0.001**	*p* = 1.000

Values are reported as mean and standard deviation. Statistical differences between groups are in **bold**.

**Table 4:** Correlation between changes in empathy over time and cerebral hypometabolism in in logopenic primary progressive aphasia and amnesic Alzheimer’s disease patients at 18F-FDG-PET SPM analysis.

**Negative Correlation between ∆PT (PT-T0 - PT-T1) and Brain Metabolism**						
	**Cluster Extent**	**Talairach Coordinates (mm)**			**T Score**	
		**x**	**y**	**z**		
**Amnesic AD**						
R Superior Temporal Gyrus	130	32.0	16.0	−24.0	4.90	
R Middle Frontal Gyrus	90	24.0	18.0	43.0	3.69	
R Fusiform Gyrus	67	57.0	−43.0	−8.0	3.38	
**lv-PPA**						
L Inferior Parietal Lobule	138	−30.0	−50.0	45.0	4.95	
L Superior Frontal Gyrus	125	−8.0	−1.0	66.0	4.83	
L Middle Frontal Gyrus		−10.0	−11.0	58.0	3.15	
R Posterior Paracentral Lobule	156	20.0	−42.0	56.0	4.74	
R Superior Frontal Gyrus	68	10.0	24.0	52.0	4.48	
L Insula	64	−38.0	14.0	10.0	4.13	
L Insula		−38.0	22.0	12.0	4.11	
**Positive Correlation between ∆PD (PD-T0 - PD-T1) and Brain Metabolism**						
	**Cluster Extent**	**Talairach Coordinates (mm)**			**T Score**	
		**x**	**y**	**z**		
**Amnesic AD**						
R Inferior Frontal Gyrus	121	42.0	7.0	33.0	5.43	
**lv-PPA**						
L Inferior Parietal Lobule	85	−50.0	−31.0	33.0	6.77	
R Superior Frontal Gyrus	68	14.0	−12.0	67.0	4.94	
L Insula	168	−44.0	4.0	5.0	4.93	
R Precuneus	158	14.0	−48.0	56.0	4.51	
L Superior Frontal Gyrus	77	−12.0	−14.0	67.0	4.42	
L Superior Frontal Gyrus		−6.0	−6.0	67.0	3.36	

Abbreviations: AD, Alzheimer’s disease; lv-PPA, logopenic primary progressive aphasia; L, left; R, right. Significant differences at *p* < 0.005 in both amnesic AD and in lv-PPA for negative correlation with ∆PT. Significant differences at *p* < 0.001 in amnesic AD and *p* < 0.005 in lv-PPA for positive correlation with ∆PD.

## Data Availability

The data that support the findings of this study are available from the corresponding author upon reasonable request.
